# Molecular Dynamics Simulation of Self-Assembly Processes of Diphenylalanine Peptide Nanotubes and Determination of Their Chirality

**DOI:** 10.3390/nano13131905

**Published:** 2023-06-21

**Authors:** Vladimir Bystrov, Ilya Likhachev, Sergey Filippov, Ekaterina Paramonova

**Affiliations:** Institute of Mathematical Problems of Biology—Branch of Keldysh Institute of Applied Mathematics, RAS, 142290 Pushchino, Russia; ilya_lihachev@mail.ru (I.L.); fsv141@mail.ru (S.F.); ekatp11@gmail.com (E.P.)

**Keywords:** molecular dynamics method, controlled molecular dynamics, self-assembly of nanostructures, nanotubes, diphenylalanine, chirality, dipole moment, visual differential analysis

## Abstract

In this work, we further developed a new approach for modeling the processes of the self-assembly of complex molecular nanostructures using molecular dynamics methods; in particular, using a molecular dynamics manipulator. Previously, this approach was considered using the example of the self-assembly of a phenylalanine helical nanotube. Now, a new application of the algorithm has been developed for implementing a similar molecular dynamic self-assembly into helical structures of peptide nanotubes (PNTs) based on other peptide molecules—namely diphenylalanine (FF) molecules of different chirality L-FF and D-FF. In this work, helical nanotubes were assembled from linear sequences of FF molecules with these initially different chiralities. The chirality of the obtained nanotubes was calculated by various methods, including calculation by dipole moments. In addition, a statistical analysis of the results obtained was performed. A comparative analysis of the structures of nanotubes was also performed using the method of visual differential analysis. It was found that FF PNTs obtained by the MD self-assembly method form helical nanotubes of different chirality. The regimes that form nanotubes of right chirality D from initial L-FF dipeptides and nanotubes of left chirality L from D-FF dipeptides are revealed. This corresponds to the law of changing the sign of the chirality of molecular helical structures as the level of their hierarchical organization becomes more complicated.

## 1. Introduction

The development of new approaches in the field of molecular dynamics simulation (MDS) methods is promising and practically important for many modern scientific directions. Among these, the study of the fundamental processes of the self-organization and self-assembly of molecular structures is of great interest. As is known, the self-assembly of amino acids and short peptides is the basis for the formation of many complex biomolecular structures.

The self-assembly of amino acids and peptides into complex and highly symmetrical molecular nanostructures (spheres, layers or ribbons, nanocrystals, nanotubes, etc.) has recently been the focus of attention of many researchers [1,2,3,4,5,6] since it has deep fundamental significance for studying the basic principles of the self-organization of the structures of biomacromolecular living systems. In addition, it is of great practical importance for many applications in nanotechnology and nanobiomedicine. Diphenylalanine (FF) peptide nanotubes (PNTs) are one of the characteristic and already well-studied examples of the objects that form helical tubular nanostructures [6,7,8,9,10,11,12,13,14,15,16,17]. Their self-assembly, features, and properties were studied both experimentally [8,9,10,11,17] and theoretically [12,13,14,15], including methods of X-ray diffraction analysis [16] as well as various methods of computer simulation [2,7,12,13,14,15,17]. It was shown that these nanotubes demonstrate noticeable and fundamental differences in their chiral properties: self-assembled nanotubes based on dipeptides of the left chirality L-FF were formed into helical nanotubes with a right-handed chirality D (with a right-handed screw of the helix), and those based on a right-handed chirality D-FF were formed into nanotubes of left chirality L (with a left helical screw). This turns out to be exactly in full accordance with the previously established general pattern for the change in the type of chirality upon transition to a higher level of hierarchical organization of biomacromolecular structures [18].

The assembly of diphenylalanine nanotubes is a complex process that requires certain knowledge, skills, equipment, and conditions. Diphenylalanine nanotubes (FF-PNTs) are nanostructures composed of diphenylalanine molecules that form a special tubular helical structure.

The helical symmetry of FF PNTs generates in them a significant internal polarization [7,12,13,14,15,17] and endows them with a unique combination of mechanical [17,19], electronic [12,13,20], and optical [21,22,23,24] properties, as well as a strong piezoelectric effect [7,14,25,26,27] and photoelectronic properties [28,29] useful for various applications.

Thus, diphenylalanine nanotubes are one-dimensional nanomaterials with unique properties that can be used in various fields of science and technology. Their use can also lead to new and improved electronic, optical, biomedical, and energy applications that can significantly improve our lives and make them more convenient and efficient. In addition to experimental studies, computer simulation methods are also actively used to study the structure and properties of these nanotubes.

The development and creation of new computer technologies, including the use of various methods of molecular modeling and molecular dynamics (MD), is a very promising and important direction in the study of such complex biomacromolecular nanostructures and peptide nanotubes. This is also necessary both from the point of view of studying the fundamental processes of the self-organization and self-assembly of molecular structures and their potential practical applications.

Previously, we already considered the application of computer simulation methods to the study of peptide nanotubes of different chirality [7,12,13,14,15,28,29]. The formation of a helical structure of a peptide nanotube (PNT) based on phenylalanine (F/Phe) was also considered using MDS methods [30,31]. It is known that, based on these amino acids, nanotubes of the F PNT type are also formed [32,33], as well as dipeptides and diphenylalanine (FF/(Phe)_2_), which, in turn, self-assemble into peptide diphenylalanine nanotubes [7,8,9,10,11,12,13,14,15,16,19,21,22,23,24,25,26,27,28,29]. Note that, for modeling all such molecular structures, a convenient computer tool is the HyperChem 8.0 software package [34], which we also widely used in our studies.

In this paper, we considered one of these new approaches, namely the so-called molecular dynamics (MD) manipulator, which allows for an efficient assembly of simulated molecular structures, taking into account the chirality of molecular components, using external force influences. It is the controlled MDS type—a molecular dynamic manipulator (MD manipulator). It is an imitation of the operation of a device by applying force to the existing initial structure in order to obtain a new final structure with the same chemical composition but different geometry (topology).

To demonstrate the possibility of the self-assembly of a diphenylalanine nanotube, the method of controlled molecular dynamics was used in this work. Here, controlled MD are understood as the inclusion of additional external forces that bring the linear structure to a helical conformation. At the same time, nowhere in the algorithm is it predetermined which structure—left-handed or right-handed—will be the result. A similar algorithm was also used by us in [30,31].

In this paper, an algorithm was developed and the self-assembly of a diphenylalanine nanotube was carried out based on a linear set of diphenylalanine (Phe-Phe)_n_ (or FF_n_) molecules. For this, we used one of the approaches of the molecular dynamics method, called the MD manipulator [30,31], based on the use of the MD package PUMA-CUDA [35,36], similar to how it was carried out in our earlier work on modeling the self-organization of a set of phenylalanine (Phe)_48_ (or F_48_) molecules into a helical tubular structure [30,31].

In this work, we considered the cases of both initial sets of dipeptides based on the different chirality of L-FF and D-FF. It is important here that, on the basis of dipeptides L-FF (left-handed chirality) during such self-assembly, mainly nanotubes with right-handed chirality (or with right-handed screw) D are formed, and, on the basis of D-FF (dipeptides with initially right-handed chirality), nanotubes with left-handed screw L are formed. This is shown in the calculations of chirality by various methods (including calculations by dipole moments), as well as in the statistical processing of the results of repeated MD runs. The results obtained are also confirmed by comparing the nanostructures of these nanotubes based on diphenylalanine FF PNT—experimental (natural) self-assembled nanotubes (obtained from their X-ray data) and their helical model nanostructures (obtained here by MDS methods)—using the visual-differential analysis (VDA) of biomolecular nanostructures developed by us earlier [15,37,38].

Thus, in this work, for the first time, we obtained helical nanotubes based on diphenylalanine (FF PNT) using MD modeling methods, taking into account different chiralities of their initial dipeptides (L-FF and D-FF). The final chirality of the obtained self-assembled nanotubes has other types of chirality D and L to those of the initial dipeptides. This corresponds to the known experimental results of the natural self-assembly of such dipeptides in solutions, and also to the general pattern of changing the type of chirality with the complication of molecular structures.

## 2. Main Methods

### 2.1. Model Details and Methodology for Numerical MDS Experiments

#### Main Software Platform Used

Here, as in earlier works [30,31], we used the PUMA-CUDA software package based on the PUMA software package [35,36] developed at the Laboratory of Molecular Dynamics of the Institute of Mineral Physics and Biology of the Russian Academy of Sciences, which is capable of performing calculations in parallel mode both on personal computers, including those with graphics accelerators, and on high-performance heterogeneous computing clusters. AMBER-99 was chosen as the main force field [39]. The PUMA-CUDA program supports setting an arbitrary additional (external force) influence. The program has added the ability to create additional force effects in the form of Hooke springs with a given stiffness and nominal length. The work was carried out interactively. The ability to interactively control and make changes to the software package makes a molecular dynamics simulation program a molecular MD constructor [40]. The TAMD (molecular dynamics trajectory analyzer) software package was also used to analyze the results [41,42]. Thus, two programs on a personal computer were used here: PUMA-CUDA directly performs the simulation itself, and an additional program TAMD (molecular dynamics trajectory analyzer) [41,42] connects to PUMA-CUDA using client–server technology and visualizes the current state of the system in real-time mode. In this mode, it is easy and convenient to set various force effects (to change, for example, the stiffness of the springs) and thus obtain the results of this effect on the entire system.

### 2.2. Model Details and Force MDS Technique for Self-Assembly of a Molecular Structure

#### 2.2.1. Initial Structure

The nanotube is assembled from monomers of dipeptides, Phe-Phe pairs (or, in another designation, FF). Each pair consists of two phenylalanine molecules connected by a peptide bond and located (approximately) perpendicular to each other. There are no valence bonds between FF pairs in a linear chain of a number of FF dipeptides.

**Figure 1:**
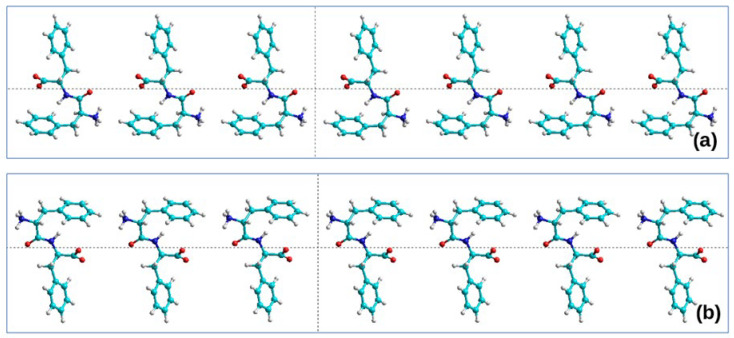
Initial linear structure of 24 dipeptides: (**a**) Poly(L-FF)_24_; (**b**) Poly(D-FF)_24_. Fragments of the 7 dipeptides are shown. Original models are constructed in the HyperChem program [34].

As a basis, a *.pdb file is taken from 24 Phe-Phe (24 FF) diphenylalanine molecules arranged linearly in a row—Poly(Phe-Phe)_24_ or Poly(FF)_24_. Such a set of molecules can be prepared using a molecular constructor of chemical structures; for example, using the HyperChem 8.0 [34] or PyMOl 2.5.4 [43] software. Thus, a structure was built from a set of 24 pairs of FF amino acids of phenylalanine F of left chirality L: here, the missing oxygen atoms O and two hydrogen atoms 2H were added to the F molecules—as a result, the structure of diphenylalanine of left chirality L-FF was obtained in the zwitterionic form. Further, this structure was replicated 24 times with a gap of 10 Å, yielding the initial linear chain Poly(L-FF)_24_ (Figure 1a). Similarly, the structure of Poly(D-FF)_24_ was constructed on the basis of phenylalanine F molecules of right chirality D—and, accordingly, diphenylalanine D-FF (Figure 1b).

In this MD simulation, chains of 24 FF molecules (L-FF/D-FF) were used; that is, a total of 43 × 24 = 1032 atoms (the diphenylalanine molecule has 43 atoms: 3 atoms from 1 H_2_O water molecule and 40 = 2 × 20 phenylalanine atoms, which create zwitterionic form of diphenylalanine). It is required to obtain a helix of 6 FF molecules per 1 coil of the helix, i.e., 6 × 43 = 258 atoms is the number of atoms per 1 coil. In total, 4 turns of 6FF molecules from 1032 atoms were formed (for both types of chirality). To prevent the molecule from moving in space, the first atom was fixed (PUMA-CUDA allows for fixing the coordinates of the necessary numbers of atoms). We added two types of springs to the force field. We used this in the same way as described in the previous work [30,31].

#### 2.2.2. The Process of Self-Assembly of the Helix

The PUMA-CUDA software package was used here as a molecular dynamics simulation program. The authors of the work, being the authors of the program, can set additional force effects. The process can be characterized as controlled molecular dynamics, and this is the MD manipulator. In this case, for the self-assembly of a diphenylalanine nanotube FF PNT, the following additional force actions were chosen (similar to [30,31]): (1) springs duplicating contacts of NH_3_ and CO_2_ groups, (2) extensions (springs) on the inner diameter, (3) extensions (springs) on the outer diameter (separate for even and odd amino acids), (4) extensions per helix pitch.

The specific lengths of the springs at rest do not matter. They are there to direct the system to the required position, not to provide a strict structure. Internal stretch marks are taken a little less (by 2 Å) than desired, and external stretch marks are taken a little more. The Hooke spring constant varies from 0.0001 pN/Å to 10 pN/Å.

The simulation was carried out in vacuum using a collisional thermostat, the parameters of which (λ = 1, ..., 10 ps^-1^ and m_0_ = 1 a.e.m.) are close to the viscosity of the aqueous environment. The temperature was chosen to be 300 K. The numerical integration step was 0.001 ps.

During the first interactive run, the values and durations of force actions were selected based on the visual representation of the structure. Further, the molecular dynamics simulation program was run 32 times under the same external influences. The implementations differed only in the sequence of the random number generator used in the implementation of the collision thermostat. The stiffness of the springs of external force actions varied as follows: 0.001 pN/Å for 5 ps, 0.01 pN/Å for 15 ps, 0.1 pN/Å for 30 ps, 1.1 pN/Å for 30 ps.

To assemble a helix from phenylalanine F, in a previous work, we gradually increased the stiffness of the spring, and there, after 90 ps of simulation, the linear structure was transformed into a nanotube [30,31]. After that, springs were added to provide additional diametrical stretching of amino acid residues (the force is applied to the terminal hydrogen atom). This was necessary to obtain a clear structure so that all residues were located on the outer side of the helix [30].

**Figure 2:**
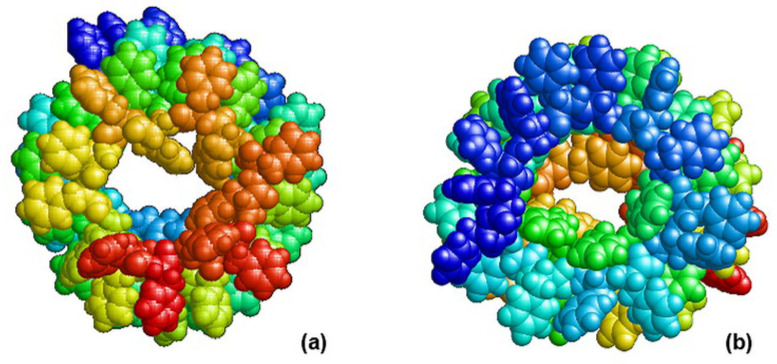
Final results after MDS self-assembly and helix structure formation: (**a**) on the left, a left-handed chiral L-form based on right-handed molecules (D-FF)_24_; (**b**) right-handed chiral D-form based on left-handed molecules (L-FF)_24_. Projection along the axis of the nanotube in the RasMol image—pdb file, using PyMOL [43].

However, in this case, it was possible to start relaxation without additional force effects. It is interesting to note that, at 30 ps (possibly less), bonds between CO_2_ and NH_3_ groups formed by themselves, which is where the initial self-assembly truly takes place. Then, during MDS (single MD run), helical structures of different chirality (right L and left D) are gradually formed depending on the set of dipeptide molecules with the initial chirality L-FF or D-FF (Figure 2).

Thus, as a result of MD modeling of the self-assembly process, with dipeptide monomers of different initial chirality L-FF (L-PhePhe) and D-FF (D-PhePhe) under the same external influences in the process of self-assembly by the MD manipulator method with a certain and sufficiently large probability, we obtained helical nanotubes of the following chirality:

right-handed helical nanotubes (D) from L-monomers (L-FF) and
left-handed helical nanotubes (L) from D-monomers (D-FF).

It should be noted that, under the same force impacts, the stability of the structures practically did not change. Nevertheless, we performed here an additional test on the stability of self-assembled FF helical structures. After conducting steered molecular dynamics, we turned off additional force effects. As a result, in this case, we obtained a series of structures under study and made sure that their helix structure remained stable. Only some free fluctuations of residues bordering the helix on one side were also observed. However, the basic helix structure was stably preserved.

However, with each new MDS run, the process follows a different MD trajectory, determined by the stochastic properties of the MD-thermostat, and not in all cases is a good orderly organized helical structure with a well-defined chirality formed. There is a certain spread and statistical fluctuations in the process of MDS formation and self-assembly of such a molecular structure.

#### 2.2.3. MD Statistics of Nanotube Assembly

To collect statistics, the computational experiment was repeated many times for each type of chirality: L and D. As a result, 32 independent realizations of assemblies of diphenylalanine nanotubes with the same initial coordinates of the system were carried out. The parameters of force impacts in all MD experiments were the same, including the strength and duration of impacts. The only thing that changed was the sequence of the random number generator, which is used by the collisional MD thermostat when playing random collisions with virtual particles. The results obtained are shown in Table 1. Comparison with other data and detailed discussion are in Section 3 below.

### 2.3. Calculation and Determination of Chirality

**Figure 3:**
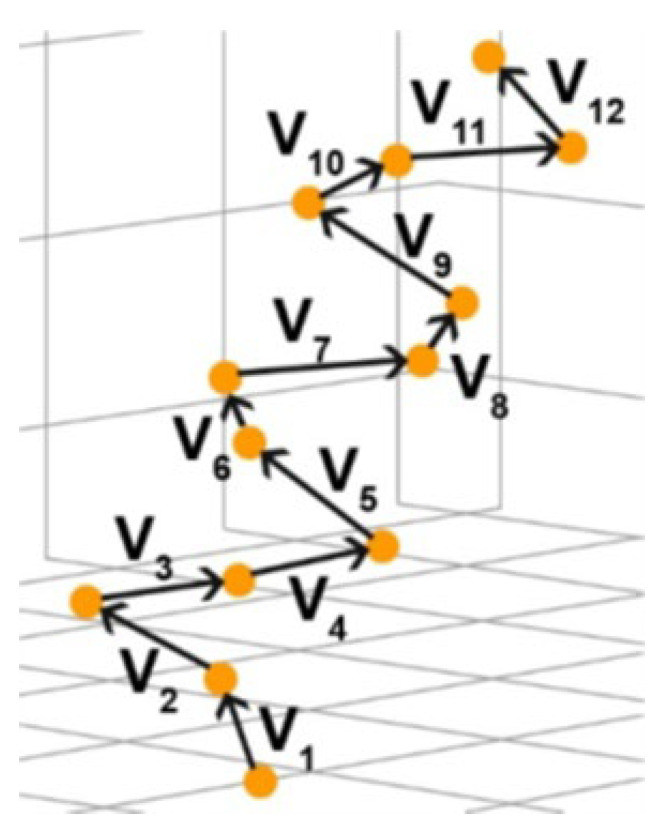
Scheme for determining the vectors of the helical structure from the references [13,44]. Cα-atoms for calculating the chirality from the mixed vector product [13].

To determine the chirality of the structure, an algorithm based on the mixed product of vectors proposed in [18,44,45] was used here. The vectors connecting the Cα-atoms of the system acted as support vectors (Figure 3). For each of the three vectors, their mixed product was found:

(1) $$\left(\left[V_{1},V_{2}\right],V_{3}\right)=\left(y_{1}z_{2}-y_{2}z_{1}\right)x_{3}+\left(z_{1}x_{2}-z_{2}x_{1}\right)y_{3}+\left(x_{1}y_{2}-x_{2}y_{1}\right)z_{3}$$

where *V*_1_, *V*_2_, *V*_3_ are vectors connecting pairs of Cα atoms.

Then, the sum of all such products was found:

(2) $$X_{\mathrm{total}}=\sum_{i=1}^{n-3}\left(\left[V1,V2\right],V3\right)$$

If this sum is positive, then the helix is right-handed and its chirality is D. If it is negative, then the helix is left-handed and its chirality is L.

For the convenience of estimating the chirality value of helical structures, a normalized value of the chirality value was also introduced [44,45]:

(3) $$\chi_{\mathrm{norm}}=\sum_{i=1}^{n-3}\frac{\left({\left[v\right]}_{i}{,v}_{i+1}{,\;v}_{i+2}\right)}{C_{i}}$$

where the normalization factor is calculated as $C_{i}={\left(\frac{1}{3}\sum_{j=0}^{2}\left|v_{i+j}\right|\right)}^{k}$, where *k* = 5.

The results of the calculation of chirality by this method for the obtained helical structures are also given in Table 1. The analysis and comparison of the results are given in Section 3.

### 2.4. Calculation of Chirality from the Mixed Product of Vectors of Dipole Moments

In this section, to determine the chirality of the helical structure, we use a method based on the mixed product of dipole moment vectors *D****_i_*** of individual dipeptides that form the helical structure of a nanotube. This method was proposed by us in [13,31]. Here, we also developed a new calculation algorithm that has a greater physical meaning. In contrast to the algorithm described above, in this case, the scalar triple-product dipole moments *D_i_* of successive individual FF molecules that make up the coils of a helical PNT nanotube were used. The origin of vectors *D****_i_*** was taken relative to the center of mass of the corresponding molecules. The absolute value of each dipole moment *D_i_*is

(4) $$D_{i}=\left|D_{i}\right|=\sqrt{D_{x,i}^{2}{+D}_{y,i}^{2}{+D}_{z,i}^{2}},$$

where *D_x,i_*, *D_y,i_*, and *D_z,i_* are the components of the *i*-th vector *D_i_* in the Cartesian coordinates.

According to [26,27,28], here, the sum of mixed (vector–scalar) products of dipole moments associated with the chirality of the PNT can be written as:

(5) $$c_{\mathrm{total}}=\sum_{i=1}^{n-2}\left(\left[D_{i}{,D}_{i+1}\right]{,D}_{i+2}\right),$$

It is necessary to note that the summation here is taken over *i* in the range from 1 to n-2, and *n* = 6 for one coil of FF PNT. However, now, we developed our algorithm for any value of n, which is explained below. The value of *c*_total_ can be normalized to the cube of the average value of the total dipole momentum of the PNT coil, $D_{\mathrm{av}}=\frac{1}{n}\sum_{i=1}^{n}D_{i}$, to obtain the universal measure of chirality:

(6) $$c_{\mathrm{norm}}=\frac{c_{\mathrm{total}}}{D_{\mathrm{av}}^{3}}.$$

Further, in this work, we developed an additional special algorithm that allows us to calculate each individual selected dipole moment of the FF dipeptide, leaving it surrounded by all other molecules of the helical structure of the nanotube. This makes it possible to obtain a more accurate calculation result, taking into account the interaction of the selected dipeptide with all other FF PNT molecules.

To build this calculation algorithm, a special **Tcl/Tk** script was developed. This script, based on the **TCL Tool Command Language** system, is embedded in Hyperchem’s options system [34].

The Hyperchem’s special **Chemist’s Developer Kit (CDK)** allows one to perform this [34]. The constructed algorithm makes it possible to select any number *n* of dipeptides and to carry out calculations not only for one coil (turn) but for any number of them, in the case of a more complex structure of the FF PNT. This is precisely the case for the FF PNT obtained here by the MD method.

**Figure 4:**
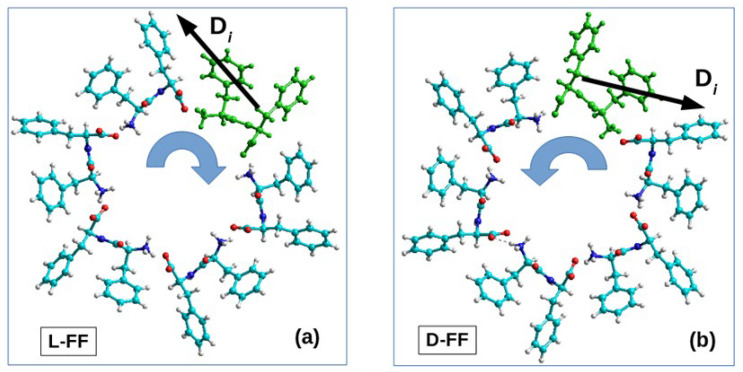
Images of helix coils of helical structures obtained from experimental data from [13,14]: (**a**) for a helix coil based on L-FF with chirality D; (**b**) for a helix coil based on D-FF with chirality L.

For additional testing and verification, we used this new algorithm to calculate chirality of the coils of the FF PNT nanotubes considered by us earlier in the papers [13,14,15] and compared the obtained chirality values (given in Table 2). As can be seen, they are somewhat different, but the sign of the chirality index remains the same. Figure 4 also shows the structures of these turns and indicates the individual vectors of dipeptides, as well as the direction of bypassing the calculation for 3 consecutive vectors of dipole moments. Further, according to this algorithm, chirality calculations were carried out for some FF PNTs constructed by the MDS method, which had fairly significant differences in chirality (from Table 1), and compared the results.

**Figure 5:**
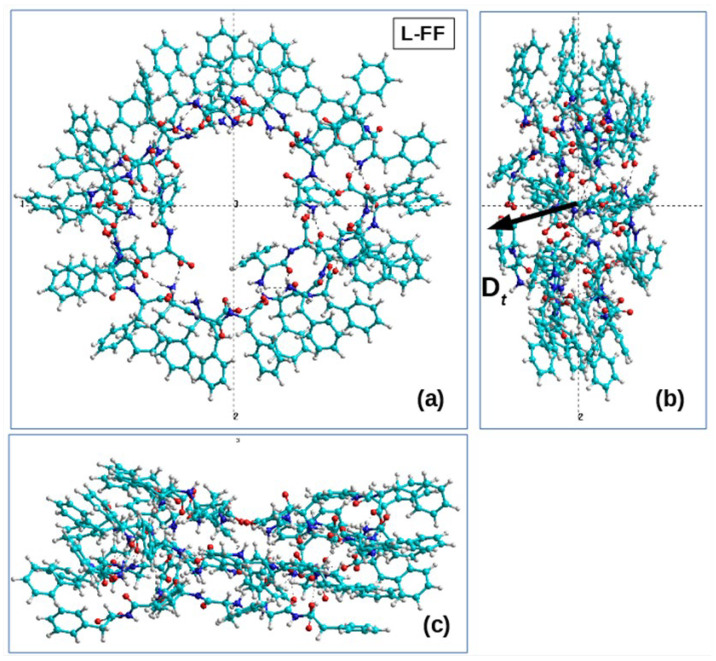
Images of the main part of the helical structure of a nanotube obtained by MDS self-assembly based on L-FF dipeptides, which has a right-handed chirality D: (**a**) view in the cross-sectional plane of a nanotube lying perpendicular to the OZ axis of the tube; (**b**) side view of the nanotube coils along the OX axis; (**c**) side view of the nanotube coils along the OY axis. The numbers in the figure mean the numbers of the coordinate axes 1-X, 2-Y, 3-Z. This structure consists of 19 FF dipeptides and is obtained on the basis of MDS-assembled nanotube No. 31 (in Table 1) formed from 24 FF dipeptides. (Here, 5 dipeptides were removed, forming poorly ordered regions at the ends of this helical structure, and its main part was left).

**Figure 6:**
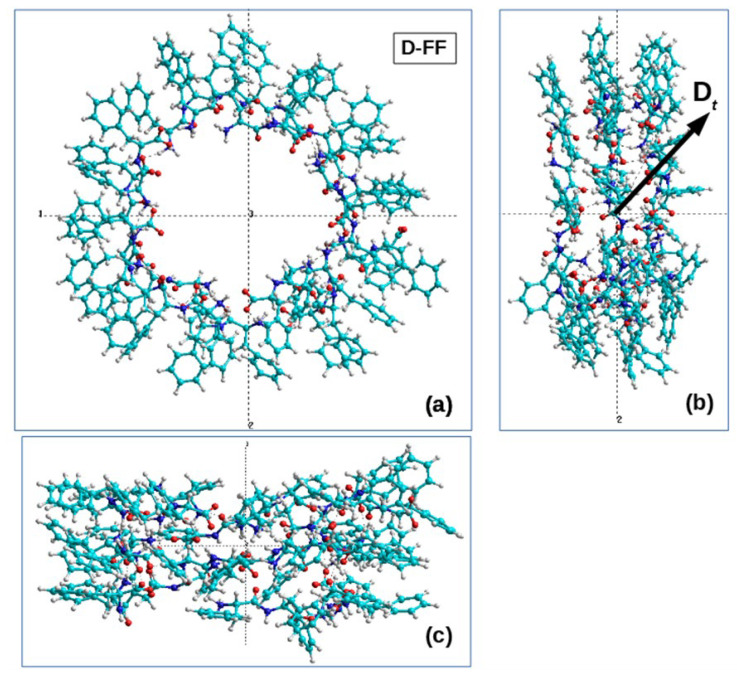
Images of the main part of the helical structure of a nanotube obtained by MD self-assembly based on D-FF dipeptides, which has a left chirality L: (**a**) view in the cross-sectional plane of a nanotube lying perpendicular to the OZ axis of the tube; (**b**) side view of the nanotube coils along the OX axis; (**c**) side view of the nanotube coils along the OY axis. The numbers in the figure mean the numbers of the coordinate axes 1-X, 2-Y, 3-Z. This structure consists of 19 FF dipeptides and is obtained on the basis of nanotube No. 25 (in Table 1) formed from 24 FF dipeptides. (Here, 5 dipeptides were removed, forming poorly ordered regions at the ends of this helical structure, and its main part was left).

In this case, by way of example, we present in Table 2 the results for the structures No. 25 for the initial D-PP dipeptide and No. 31 for initial L-FF, which are shown in Table 1. All the obtained data on the calculated values of chirality are given in the final Table 2. Figure 5 and Figure 6 also show the main coils of these MDS PNT FF structures.

The directions of the dipole moment vector *D_i_* of one of the FF dipeptides in both cases of different chirality are shown, as well as the directions of bypassing the coil of the helix when calculating the chirality value according to Formula (5) for both chirality types.

### 2.5. Methods for Analyzing and Evaluating the Reliability of the Obtained MD Structures

To assess the reliability of the results obtained, visual differential analysis (VDA) [15,37,38] of projection hypsometric maps of surfaces [38,46,47,48] for self-assembly by the MDS method and experimental diphenylalanine nanotubes data was used. From the entire set of structures obtained in the course of numerical MDS experiments, two structures were selected (one for each D and L chirality), whose configuration visually turned out to be the closest to the configuration of the experimental D and L nanotubes [14,15,37]. To obtain consistent hypsometric maps, the selected MD structures were aligned relative to the experimental ones with the corresponding chirality in the PyMol program [43] using the pair_fit command. Alignment was performed according to the nitrogen atoms of the α-amino group constituting the peptide bond. These atoms are less mobile and form a kind of nanotube backbone.

Alignment of the MD structure obtained from D-FF monomers was carried out over four pairs of atoms located at the corners of a secant rectangle lying on the vertical axis and the diameter of each of the two nanotubes—experimental and MD. The root mean square deviation of atomic positions (RMSD) was 2.8 Å. The alignment of the MD structure obtained from L-FF monomers was carried out over 21 pairs of atoms located on the surface of the conditional cylinder of the experimental and MD nanotubes. RMSD was 2.92 Å.

For four structures prepared in this way, three-dimensional molecular models [46,47,48] were built in the Blender free 3D editor [48], and projection hypsometric maps of the outer and inner surfaces of nanotubes were obtained: experimental and those obtained by the MD method, with chirality D and L. The maps were rendered “topographically” using the free digital imaging program G’MIC [49] with the **topographic_map command “30,15”**.

## 3. Main Results and Discussions

The main results of the MDS assembly of diphenylalanine nanotubes obtained from L- and D-monomers in series of 32 MDS independent runs each are shown in Table 1. The results of the calculations of chirality values for the MDS-assembled nanotubes in comparison with other data are shown in Table 2. As a result of such numerical MDS experiments, mixed cases were also obtained, where part of the structure was left-handed and the other part turned out to be right-handed. When constructing Table 1, an expert assessment was carried out, where, based on its results, such “complex” structures were recognized—twisted half to the left side and half to the right (this corresponds to the values of the number 0.5 in the corresponding columns). The results obtained by the algorithms for calculating chirality, according to Formulas (1) and (2), unambiguously recognize such structures. Therefore, there is a slight discrepancy in the recognition of structures by the method of expert evaluation by a person and using a numerical algorithm for calculating chirality (1) and (2). As the experience of carrying out such computational experiments by MD methods with a large number of implementations shows, not all monomers of chirality D yield left-handed helices, but L-monomers yield right-handed ones. The list of received helices is given in Table 1. Brief main conclusions on the obtained results of the analysis of statistics (data from Table 1) and calculations of chirality using Formulas (1)–(3) are as follows.

According to the assembly statistics obtained, right-handed nanotubes can be obtained from L-monomers with a linear structure with a probability of approximately 80%, and right-handed nanotubes can be obtained from D-monomers with approximately the same probability. Computational experiments are always limited by the main resource—the processor time. In this work, we show the very principle of assembling molecular structures using controlled molecular dynamics and show the possibility of obtaining a statistically reliable result. In order to determine the structure of the helix, we gradually increased the external force effects, bringing the linear monomer to the desired helical form.

The results obtained are also well confirmed by calculations of the chirality by another method, where, in the mixed (vector–scalar) product that determines this characteristic, vectors of dipole moments of individual dipeptides located sequentially along the helix are used. In this case, this characteristic also has a greater physical meaning.

This method is based on Formulas (4)–(6). In this work, we also built a new algorithm for calculating these formulas for any number of dipeptides and turns in a helical structure, using special options based on the **Tool Command Language TCL** system built into the Hypercham options system, which allows one to enter a special script that speeds up all calculations and ensures their higher physical reliability.

Here, for comparison, we also used the previously obtained data for the turns of the L-FF and D-FF helices constructed on the basis of experimental X-ray data for the structures of such molecular crystals grown from solutions [11,16,17]. It is known that these structures are highly ordered and have significant total dipole moments (and the corresponding polarization) directed along the nanotube axis [2,7,12,13,14,15]. Figure 5b and Figure 6b show the total dipole moments Dt for the structures taken from Table 1, constructed by the MD method and with different chiralities, confirmed by expert evaluation and by calculations using Formulas (1)–(3). These total dipole moments were calculated by the Amber and BIO CHARM methods from the HyperChem package [34], and they show that the main components of the vector Dt are oriented here along the nanotube axis (albeit not strictly along the axis, since, here, we also have a certain lack of order). It is also important that the directions of these vectors for the obtained L and D nanotubes are opposite, as should be for structures of different chiralities.

All the main calculations of the dipole moments of individual dipeptides in our helical structures (and, accordingly, the calculations of chirality based on them, given in Table 2) were carried out by semi-empirical quantum chemical PM3 methods in the restricted Hartree–Fock (RHF) approximation from the Hypercham package [34]. As can be seen from the results presented in Table 2, the chirality values of the considered nanotubes constructed by the MD method calculated in this way using Formulas (4)–(6) are in very good agreement in magnitude and agree in sign with known and well-ordered helical nanostructures.

As can be seen from Table 2, the results obtained here, as well as those previously obtained in [13,14,15,17], show a characteristic change in the sign of chirality upon transition to a higher level of organization, which is observed in the structures of biomacromolecules [18]: the calculated chirality of a helical nanotube based on dipeptide L-FF has a positive sign—D-type—and the chirality of the nanotube based on the D-FF dipeptide has a negative sign, corresponding to the L-type chirality (see Table 1).

Thus, the PNT FFs assembled by the MD method demonstrated the same chirality and its changes as the previously experimentally obtained PNT FF.

## 4. Results of Comparative Visual Differential Analysis

A visual differential analysis of external (Figures S1 and S2) and internal (Figures S3 and S4) surfaces of self-assembled structures confirms the geometric similarity of MD structures to their corresponding structures obtained in the course of a full-scale experiment [11,16,17,37]. The analysis is presented in detail in the attached materials (Supplementary Materials). The main reason is that the direction of the helical turns of the D-FF nanotubes obtained by MD calculations corresponds to the direction of the helical turns of the D-FF nanotube obtained in the course of a full-scale experiment.

The same is true for L-FF nanotubes: the direction of the turns of the helix obtained in the course of MD calculations coincides with the direction of the turns of the experimental L-FF nanotube. In addition, a number of patterns are clearly visible on all maps without exception. In particular, the range of “heights” and “depths” for all MD structures is noticeably wider than that for experimental nanotubes. For example, the layer thickness of experimental nanotubes is 7.1 Å for D-FF and 7.7 Å for L-FF, which is consistent with the results obtained earlier [37], whereas the thickness of the molecular layer for self-assembled MD nanotubes is 14 Å for D-FF and 12 Å for L-FF. However, hypsometric maps show that such values are not ubiquitous and are only due to the “ejections” of individual aromatic rings of diphenylalanine monomers outside (Figures S1 and S2) or inside the nanotube channel (Figures S3 and S4). Thus, the average values of distances (radii) from each atom of a nanotube to its axis are more indicative (Table 3).

It can be seen from Table 3 that, although the diameter of self-assembled MD nanotubes is noticeably larger (by ~3.5 Å) than that of experimental nanotubes, there are practically no differences between the molecular dynamic D- and L-structures in this parameter. 

However, it can be noted here that, for example, in [17], it was shown that nanotubes based on L-FF and D-FF, grown simultaneously and under the same experimental conditions, have different crystallographic space groups, have differences in size, and demonstrate different growth kinetics. Thus, nanotubes based on D-FF turned out to be thicker and shorter than those based on L-FF, but this is already a bundle of nanotubes in [17], whereas individual nanotubes are characterized by shorter distances between the turns of D-FF nanotubes in the direction of the a axis (in the crystallographic structures of nanotubes) compared to the turns for L-FF nanotubes: approximately a ~23.8 Å for D-FF and a ~24.8 Å for L-FF (in the experimental cases obtained there) [7,17].

## 5. Conclusions

Thus, this work shows the fundamental possibility of the self-assembly of diphenylalanine peptide nanotubes (FF PNTs) by methods of controlled (steered) molecular dynamics, similar to the case of the previously performed self-assembly of the phenylalanine peptide nanotube (F PNT) [30,31] using the MD manipulator approach with suitable modifications. Moreover, it is important that here, on the basis of dipeptides of left chirality L-FF, nanotubes of right chirality D are formed, and, on the basis of dipeptides D-FF, helical nanotubes of left chirality L are formed. This corresponds to the pattern of changing chirality with the hierarchical complication of biomolecular structures.

It should be noted that these issues of the self-organization and self-assembly of peptide nanostructures and, in particular, peptide nanotubes based on diphenylalanine dipeptides, are the focus of many research groups, due, as already noted in the introduction, to the unique physical properties of these nanotubes, which are attractive for their various applications. Due to the limited scope and narrower focus of this work, we cannot cover the entire scope of ongoing research.

Nevertheless, in conclusion, we would like to note some works that may have a closer connection with this work of ours or may have some prospect for the further continuation of research in this direction, particularly such known works as [3,4,5,6,7,8,9,10,11,20,21,33,50,51,52] and [53], where molecular dynamics (MD) simulations of FF in water and methanol solvents have been performed using an explicit solvent model. These works are very interesting and could be further developed using our new MD method.

The various productive approaches of MD in the works on the self-assembly of diphenylalanine structures should also be noted [54,55]. However, coarse-grained molecular dynamics (MD) simulations were used in [54], whereas we used a full-atomic model. Here, we believe that we are out of competition. In [55], another interesting PACE hybrid model was used, with which it is difficult to directly compare our results.

There are also some very interesting works in this area of the Marchesan group, particularly a recent good overview of various dipeptide sequences (including FF) [56], which have been reported in various publications, for their ability to form nanotubes, which often have water-filled supramolecular channels, as well as works that considered the self-assembly of dipeptides in not only nanotubes but also gel [57]. Here, it is interesting that our studied phenylalanine molecules [30,31] can also self-assemble into hydrogels and then also self-assemble into crystals. This still needs to be investigated separately and further. However, here, it is already clear that hydrophobicity helps them in this self-assembly, as similarly reported in [57].

This work also shows not only the applicability of this algorithm for assembling helical peptide nanotubes to diphenylalanine, in addition to phenylalanine (as was carried out in previous works [30,31]), but also, thereby, the possibility of using such an MD method for assembling helical peptide structures based on any other dipeptides; in particular, for example, for dileucine and diisoleucine [2,11,58,59].

In addition, the approach developed here for calculating the chirality of helical peptide structures can also be further applied to other similar structures based on dipeptides, particularly to estimate the magnitude and sign of the chirality of helical structures based on the same dileucine (LL) mentioned above—left L-LL and right D-LL—as well as diisoleucine (II)—left (L-II) and right (D-II). These promising works are already being actively developed and new results can be expected here soon.

Finally, the results obtained here are also confirmed by a statistical analysis of the data on the conducted MD runs (computational experiments) and calculations of the chirality index based on two methods of the mixed vector–scalar product of vectors constructed from Ca atoms of dipeptides and on vectors of dipole moments of individual dipeptides, as well as by a comparison and visual differential analysis of the obtained helical MD structures of different chirality with experimentally self-assembled diphenylalanine peptide nanotubes. Of course, in the obtained results of MD self-assembly, a certain scatter of the data obtained is observed, and some MD runs lead to not quite correct structures. However, these results are a consequence of the quite obvious statistical nature of this approach, where each individual MD run generates its own random set of interactions in the collisional thermostat used in this MD method. All this also leads to the formation of a possibly not-well-ordered structure of nanotubes, which differs from the structures of nanotubes obtained under experimental conditions of their self-assembly. Note that this difference between them is well revealed by the VDA method, which has shown its effectiveness here.

We also note that, in this MD approach, a sort of simplified medium is modeled, in which these helical nanotubes are formed by simulating its effects using the applied parameters of “Hooke’s springs” (longitudinal and transverse). Nevertheless, it is important that, under the same conditions, starting dipeptides of the same chirality form helical nanotubes of another chirality, corresponding to the established pattern of changing the type of chirality. This is a fundamentally important principle—and is shown here by MD self-assembly methods.

## Figures and Tables

**Table 1:** Statistics of obtained chirality (value and sign) by assembled FF helix structures.

Run Number	L-Monomer			D-Monomer		
	Number of Left Helices	Number of Right Helices	Value According to the Formulas (1)–(3)	Number of Left Helices	Number of Right Helices	Value According to the Formulas (1)–(3)
1		1	−0.006	0.8	0.2	−0.046
2		1	0.033	1		−0.098
3		1	0.009	0.2	0.8	−0.031
4		1	0.043	0.8	0.2	−0.045
5		1	0.005	1		−0.001
6	0.5	0.5	−0.032	0.5	0.5	−0.007
7		1	0.018	1		−0.030
8	1		−0.027		1	−0.031
9	1		−0.038	1		−0.023
10	0.5	0.5	0.042	1		−0.002
11	1		0.008	0.5	0.5	0.050
12		1	0.016	1		−0.027
13	0.5	0.5	0.062	0.5	0.5	0.004
14		1	0.001	0.5	0.5	0.003
15		1	0.039	0.5	0.5	0.018
16		1	0.031	1		−0.003
17	0.5	0.5	0.040	**1**		0.004
18		1	0.003	0.5	0.5	−0.028
19		1	−0.004	1		−0.043
20	0.5	0.5	0.015	0.8	0.2	−0.063
21		1	0.073	0.5	0.5	0.003
22		1	0.050	0.2	0.8	−0.054
23		1	0.059	0.5	0.5	−0.030
24		1	0.054	0.5	0.5	−0.029
25		1	0.048	1		−0.022
26		1	0.015	1		−0.011
27	0.5	0.5	0.044	0.5	0.5	−0.039
28	0.5	0.5	0.086	0.5	0.5	0.012
29	0.5	0.5	0.003	0.8	0.2	−0.012
30		1	−0.021	0.5	0.5	−0.011
31		1	0.043	0.5	0.5	−0.007
32		1	0.034	0.8	0.2	−0.016
**Total expert opinion**	7 (22%)	25 (78%)		21.9 (68%)	10.1 (32%)	
**Total according to** Formulas (1)–(3)	6 (19%)	26 (81%)		25 (78%)	7 (22%)	

**Table 2:** Magnitudes and signs of the chirality values obtained for L-FF and D-FF PNTs, obtained by PM3 RHF method using HyperChem package (in normalized form *c*_norm_ (6)).

Initial Chirality Type of the FF Molecule	L-FF	D-FF
Computed data for 6FF PNT coil from [14,15]	1.219	−0.674
Computed data for 6FF PNT coil in present work	1.357	−1.347
Computed data for 19FF PNT obtained by MD method in present work	0.7771	−1.2302
Chirality PNT sign	Positive	Negative
Chirality PNT symbol	D	L

**Table 3:** Average distances (Å) from each PNT atom to its axis.

Experimental D-FF NT	9.08
Experimental L-FF NT	9.17
Molecular dynamics D-FF NT	10.89
Molecular dynamics L-FF NT	10.87

## Data Availability

The data presented in this study are available on request from the corresponding author.

## Supplementary Materials

The following supporting information can be downloaded at: https://www.mdpi.com/article/10.3390/nano13131905/s1, Colour images for visual differential analysis (VDA). Figure S1. Parallel representation of hypsometric maps of the outer surface of self-assembled nanotubes with chirality D, obtained in the course of natural (top) and MDS numerical (bottom) experiments. Figure S2. Parallel representation of hypsometric maps of the outer surface of self-assembled nanotubes with chirality L, obtained in the course of natural (top) and MDS numerical (bottom) experiments. Figure S3. Parallel representation of hypsometric maps of the inner surface of the channels of self-assembled nanotubes with chirality D, obtained in the course of natural (top) and MDS numerical (bottom) experiments. Figure S4. Parallel representation of hypsometric maps of the inner surface of the channels of self-assembled nanotubes with chirality L, obtained in the course of natural (top) and MDS numerical (bottom) experiments.

## References

[1] Nepal D., Kang S., Adstedt K.M., Kanhaiya K., Bockstaller M.R., Brinson L.C., Buehler M.J., Coveney P.V., Dayal K., El-Awady J.A. (2023). Hierarchically structured bioinspired nanocomposites. Nat. Mater..

[2] Bystrov V. (2020). Computer Simulation Nanostructures: Bioferroelectric Amino Acids. Bioferroelectricity: Peptide Nanotubes and Thymine Nucleobase.

[3] Makam P., Gazit E. (2018). Minimalistic peptide supramolecular co-assembly: Expanding the conformational space for nanotechnology. Chem. Soc. Rev..

[4] Scanlon S., Aggeli A. (2008). Self-assembling peptide nanotubes. Nano Today.

[5] Yuan C., Ji W., Xing R., Li J., Gazit E., Yan X. (2019). Hierarchically oriented organization in supramolecular peptide crystals. Nat. Rev. Chem..

[6] Raymond D.M., Nilsson B.L. (2018). Multicomponent peptide assemblies. Chem. Soc. Rev..

[7] Bystrov V.S., Zelenovskiy P.S., Nuraeva A.S., Kopyl S., Zhulyabina O.A., Tverdislov V. (2019). Chiral Peculiar Properties of Self-Organization of Diphenylalanine Peptide Nanotubes: Modeling of Structure and Properties. Math. Biol. Bioinform..

[8] Kol N., Adler-Abramovich L., Barlam D., Shneck R.Z., Gazit E., Rousso I. (2005). Self-Assembled Peptide Nanotubes Are Uniquely Rigid Bioinspired Supramolecular Structures. Nano Lett..

[9] Shklovsky J., Beker P., Amdursky N., Gazit E., Rosenman G. (2010). Bioinspired peptide nanotubes: Deposition technology and physical properties. Mater. Sci. Eng. B.

[10] Reches M., Gazit E. (2006). Controlled patterning of aligned self-assembled peptide nanotubes. Nat. Nanotechnol..

[11] Görbitz C.H. (2001). Nanotube formation by hydrophobic dipeptides. Chem. Weinh. Bergstr. Ger..

[12] Bystrov V.S., Paramonova E., Bdikin I., Kopyl S., Heredia A., Pullar R.C., Kholkin A.L. (2012). BioFerroelectricity: Diphenylalanine Peptide Nanotubes Computational Modeling and Ferroelectric Properties at the Nanoscale. Ferroelectrics.

[13] Bystrov V., Sidorova A., Lutsenko A., Shpigun D., Malyshko E., Nuraeva A., Zelenovskiy P., Kopyl S., Kholkin A. (2021). Modeling of Self-Assembled Peptide Nanotubes and Determination of Their Chirality Sign Based on Dipole Moment Calculations. Nanomaterials.

[14] Bystrov V., Coutinho J., Zelenovskiy P., Nuraeva A., Kopyl S., Zhulyabina O., Tverdislov V. (2020). Structures and Properties of the Self-Assembling Diphenylalanine Peptide Nanotubes Containing Water Molecules: Modeling and Data Analysis. Nanomaterials.

[15] Bystrov V.S., Filippov S.V. (2022). Molecular modelling and computational studies of peptide diphenylalanine nanotubes, containing waters: Structural and interactions analysis. J. Mol. Model..

[16] CCDC Home|CCDC 16340 for FF—from Refs #11 (Gorbitz), CCDC 1853771—from Refs #17 (Zelenovskii). http://www.ccdc.cam.ac.uk.

[17] Zelenovskiy P.S., Nuraeva A., Kopyl S., Arkhipov S.G., Vasilev S.G., Bystrov V.S., Gruzdev D.A., Waliczek M., Svitlyk V., Shur V.Y. (2019). Chirality-Dependent Growth of Self-Assembled Diphenylalanine Microtubes. Cryst. Growth Des..

[18] Tverdislov V.A. (2013). Chirality as a primary switch of hierarchical levels in molecular biological systems. Biophysics.

[19] Zelenovskiy P., Kornev I., Vasilev S., Kholkin A. (2016). On the origin of the great rigidity of self-assembled diphenylalanine nano-tubes. Phys. Chem. Chem. Phys..

[20] Tao K., Makam P., Aizen R., Gazit E. (2017). Self-assembling peptide semiconductors. Science.

[21] Amdursky N., Molotskii M., Aronov D., Adler-Abramovich L., Gazit E., Rosenman G. (2009). Blue luminescence based on quan-tum confinement at peptide nanotubes. Nano Lett..

[22] Gan Z., Wu X., Zhu X., Shen J. (2013). Light-induced ferroelectricity in bioinspired self-assembled diphenylalanine nano-tubes/microtubes. Angew. Chem. Int. Ed. Engl..

[23] Gan Z., Wu X., Zhang J., Zhu X., Chu P.K. (2013). In Situ Thermal Imaging and Absolute Temperature Monitoring by Luminescent Diphenylalanine Nanotubes. Biomacromolecules.

[24] Nikitin T., Kopyl S., Shur V., Kopelevich Y., Kholkin A. (2016). Low-temperature photoluminescence in self-assembled diphenylalanine microtubes. Phys. Lett. A.

[25] Nguyen V., Zhu R., Jenkins K., Yang R. (2016). Self-assembly of diphenylalanine peptide with controlled polarization for power generation. Nat. Commun..

[26] Jenkins K., Kelly S., Nguyen V., Wu Y., Yang R. (2018). Piezoelectric diphenylalanine peptide for greatly improved flexible nano-generators. Nano Energy.

[27] Vasilev S., Zelenovskiy P., Vasileva D., Nuraeva A., Shur V.Y., Kholkin A.L. (2016). Piezoelectric properties of diphenylalanine microtubes prepared from the solution. J. Phys. Chem. Solids.

[28] Bystrov V. (2018). Photoferroelectricity in di-phenylalanine peptide nanotubes. Comput. Condens. Matter.

[29] Bystrov V., Paramonova E., Zelenovskii P., Kopyl S., Shen H., Lin T., Fridkin V. (2023). Photoelectronic Properties of Chiral Self-Assembled Diphenylalanine Nanotubes: A Computational Study. Symmetry.

[30] Likhachev I., Bystrov V. (2021). Assembly of a Phenylalanine Nanotube by the use of Molecular Dynamics Manipulator. Math. Biol. Bioinform..

[31] Bystrov V., Likhachev I., Sidorova A., Filippov S., Lutsenko A., Shpigun D., Belova E. (2022). Molecular Dynamics Simula-tion Study of the Self-Assembly of Phenylalanine Peptide Nanotubes. Nanomaterials.

[32] German H.W., Uyaver S., Hansmann U.H.E. (2014). Self-Assembly of Phenylalanine-Based Molecules. J. Phys. Chem. A.

[33] Adler-Abramovich L., Vaks L., Carny O., Trudler D., Magno A., Caflisch A., Frenkel D., Gazit E. (2012). Phenylalanine assembly into toxic fibrils suggests amyloid etiology in phenylketonuria. Nat. Chem. Biol..

[34] (2011). HyperChem 8. Tools for Molecular Modeling. Professional Edition for Windows AC Release 8.0 USB (on CD). Gainesville, FL 32601 United States: Hypercube. Inc. http://www.hypercubeusa.com/.

[35] Lemak A.S., Balabaev N.K. (1995). A Comparison Between Collisional Dynamics and Brownian Dynamics. Mol. Simul..

[36] Lemak A.S., Balabaev N.K. (1996). Molecular dynamics simulation of a polymer chain in solution by collisional dynamics method. J. Comput. Chem..

[37] Filippov S.V., Bystrov V.S. (2020). A Visual Differential Analysis of Structural Features of Internal Cavities in Two Chiral Forms of Diphenylalanine Nanotubes. Biophysics.

[38] Bystrov V.S., Filippov S.V. (2021). Computer modeling and numerical studies of peptide nanotubes based on diphenylalanine. Keldysh Inst. Prepr..

[39] Wang J.M., Cieplak P., Kollman P. (1999). How Well Does a Restrained Electrostatic Potential (RESP) Model Perform in Calculat-ing Conformational Energies of Organic and Biological Molecules?. J. Comput. Chem..

[40] Glyakina A.V., Likhachev I.V., Balabaev N.K., Galzitskaya O.V. (2018). Comparative mechanical unfolding studies of spectrin domains R15, R16 and R17. J. Struct. Biol..

[41] Balabayev N.K., Likhachev I. (2007). Trajectory Analyzer of Molecular Dynamics. Math. Biol. Bioinform..

[42] Likhachev I.V., Balabaev N., Galzitskaya O.V. (2016). Available Instruments for Analyzing Molecular Dynamics Trajectories. Open Biochem. J..

[43] PyMOL Pymol.Org. https://pymol.org/2/.

[44] Tverdislov V.A., Sidorova A.E., Bagrova O.E., Belova E.V., Bystrov V.S., Levashova N.T., Lutsenko A.O., Semenova E.V., Shpigun D.K. (2022). Chirality as a Symmetric Basis of Self-Organization of Biomacromolecules. Biophysics.

[45] Sidorova A., Bystrov V., Lutsenko A., Shpigun D., Belova E., Likhachev I. (2021). Quantitative Assessment of Chirality of Protein Secondary Structures and Phenylalanine Peptide Nanotubes. Nanomaterials.

[46] Filippov S.V. (2018). Blender software platform as an environment for modeling objects and processes of science disciplines. Keldysh Inst. Prepr..

[47] Filippov S.V. (2019). Method for the identification of atoms of macromolecules visualized in 3D-editors. Keldysh Inst. Prepr..

[48] Blender Foundation blender.org—Home of the Blender Project—Free and Open 3D Creation Software [Electronic Resource]. http://www.blender.org.

[49] G’MIC—GREYC’s Magic for Image Computing: A Full-Featured Open-Source Framework for Image Processing—Main [Electronic Resource]. https://gmic.eu/.

[50] Reches M., Gazit E. (2003). Casting Metal Nanowires Within Discrete Self-Assembled Peptide Nanotubes. Science.

[51] Adler-Abramovich L., Gazit E. (2014). The physical properties of supramolecular peptide assemblies: From building block association to technological applications. Chem. Soc. Rev..

[52] Tamamis P., Adler-Abramovich L., Reches M., Marshall K., Sikorski P., Serpell L., Gazit E., Archontis G. (2009). Self-Assembly of Phenylalanine Oligopeptides: Insights from Experiments and Simulations. Biophys. J..

[53] Rissanou A.N., Georgilis E., Kasotakis E., Mitraki A., Harmandaris V. (2013). Effect of Solvent on the Self-Assembly of Dialanine and Diphenylalanine Peptides. J. Phys. Chem. B.

[54] Guo C., Luo Y., Zhou R., Wei G. (2012). Probing the Self-Assembly Mechanism of Diphenylalanine-Based Peptide Nanovesicles and Nanotubes. ACS Nano.

[55] Xiong Q., Jiang Y., Cai X., Yang F., Li Z., Han W. (2019). Conformation Dependence of Diphenylalanine Self-Assembly Structures and Dynamics: Insights from Hybrid-Resolution Simulations. ACS Nano.

[56] Bellotto O., D’andrea P., Marchesan S. (2023). Nanotubes and water-channels from self-assembling dipeptides. J. Mater. Chem. B.

[57] Bellotto O., Pierri G., Rozhin P., Polentarutti M., Kralj S., D’Andrea P., Tedesco C., Marchesan S. (2022). Dipeptide self-assembly into water-channels and gel biomaterial. Org. Biomol. Chem..

[58] Bystrov V.S., Bdikin I.K., Singh B. (2020). Piezoelectric and ferroelectric properties of various amino acids and tubular dipeptide nanostructures: Molecular modeling. Nanomater. Sci. Eng..

[59] Bystrov V.S., Paramonova E.V., Yurkova D.O., Ledeneva O.R., Belova E.V. (2023). Photoelectronic properties of peptide nanotubes based on various amino acids. Proceedings of the VII Cogress of Biophysics of Russia.

