# Supplementary material for: Molecular Dynamics Simulation of Self-Assembly Processes of Diphenylalanine Peptide Nanotubes and Determination of Their Chirality
Source: Nanomaterials (Basel). 2023 Jun 21;13(13):1905. doi: 10.3390/nano13131905 (PMC10343799; doi:10.3390/nano13131905)
Supplement: Supplementary file 1 [file nanomaterials-13-01905-s001.zip › nanomaterials-2424985-supplementary.pdf]

nanomaterials

Supplementary Materials

Molecular Dynamics Simulation of Self-Assembly Processes of Diphenylalanine Peptide Nanotubes and Determination of Their Chirality

Vladimir Bystrov *, Ilya Likhachev, Sergey Filippov and Ekaterina Paramonova

Institute of Mathematical Problems of Biology—Branch of Keldysh Institute of Applied Mathematics, RAS, 142290 Pushchino, Russia; ilya_lihachev@mail.ru (I.L.); fsv141@mail.ru (S.F.); ekatp11@gmail.com (E.P.)
* Correspondence: vsbys@mail.ru or bystrov@impb.ru.

Colour images for visual differential analysis (VDA)

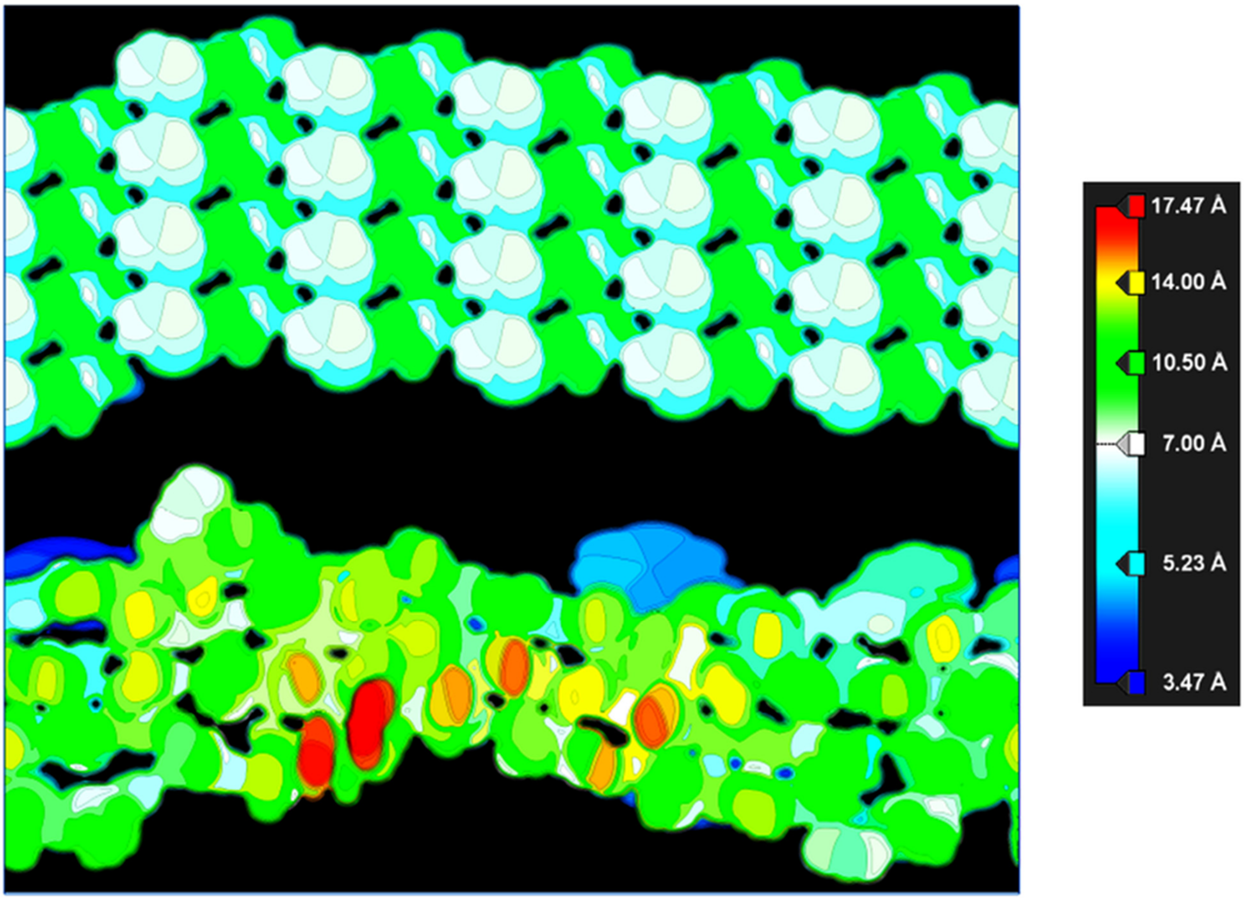

Figure S1. Parallel representation of hypsometric maps of the outer surface of self-assembled nanotubes with chirality D, obtained in the course of natural (top) and MDS numerical (bottom) experiments.

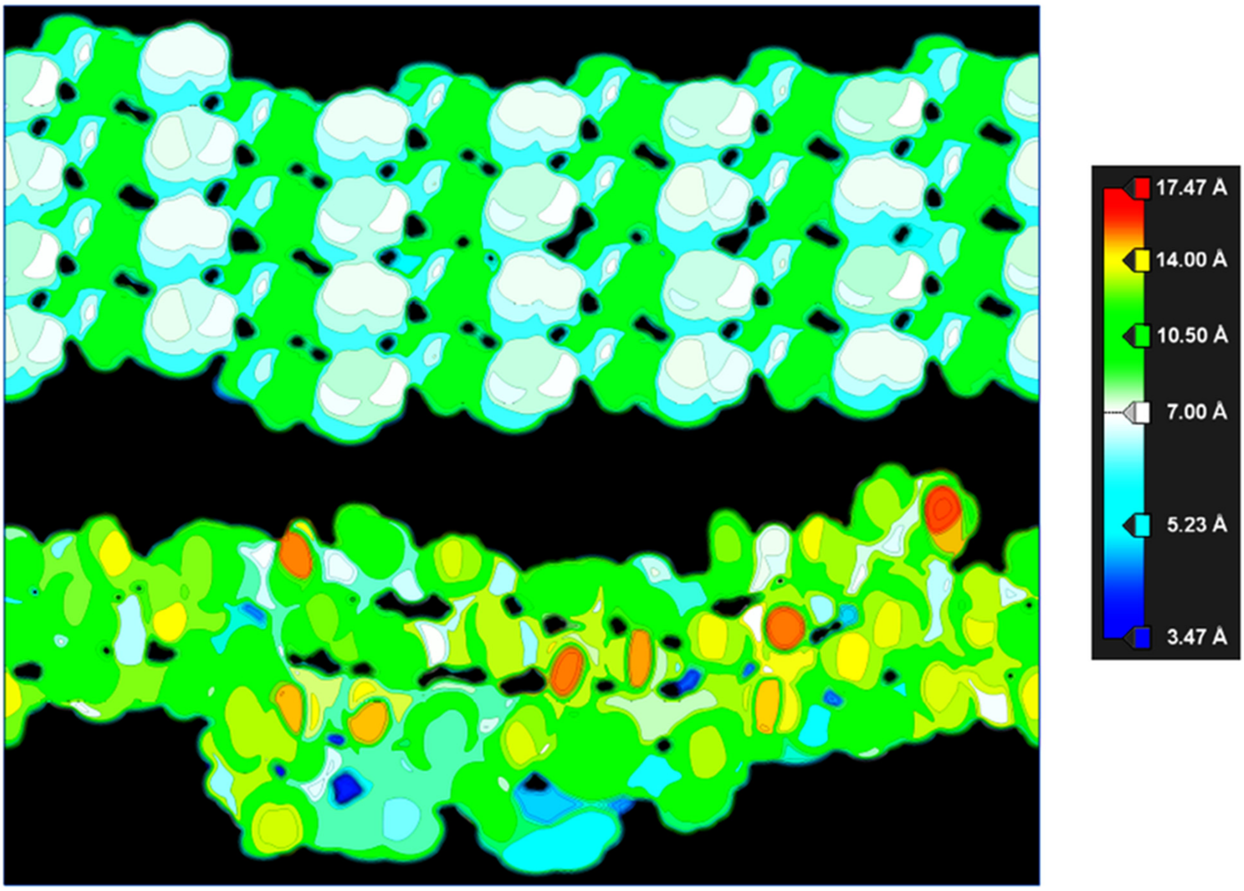

Figure S2. Parallel representation of hypsometric maps of the outer surface of self-assembled nanotubes with chirality L, obtained in the course of natural (top) and MDS numerical (bottom) experiments.

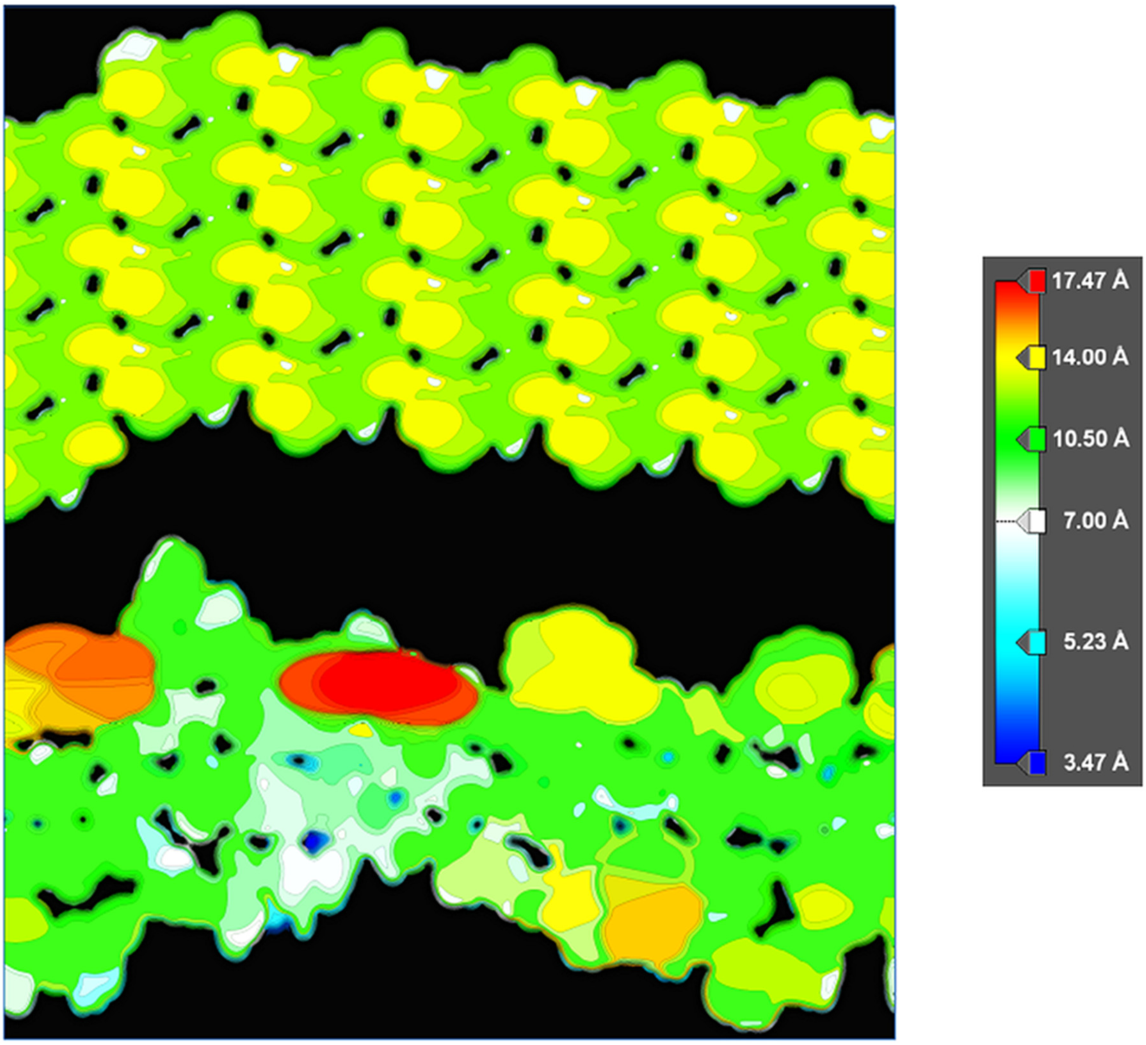

Figure S3. Parallel representation of hypsometric maps of the inner surface of the channels of self-assembled nanotubes with chirality D, obtained in the course of natural (top) and MDS numerical (bottom) experiments.

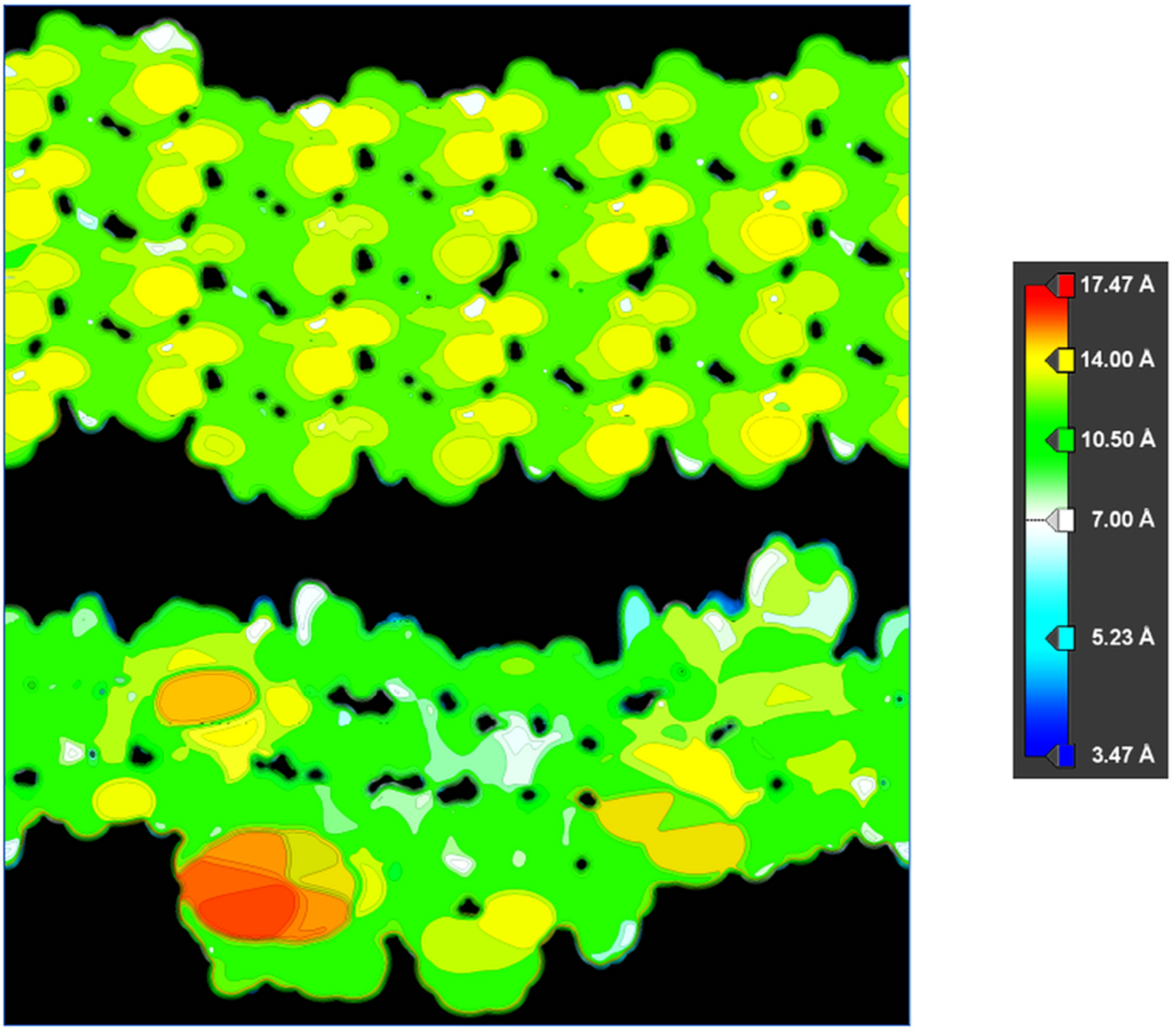

Figure S4. Parallel representation of hypsometric maps of the inner surface of the channels of self-assembled nanotubes with chirality L, obtained in the course of natural (top) and MDS numerical (bottom) experiments.
